# A mathematical framework to correct for compositionality in microbiome data sets

**DOI:** 10.1128/aem.01126-25

**Published:** 2026-01-06

**Authors:** Samuel P. Forry, Stephanie L. Servetas, Jason G. Kralj, Monique E. Hunter, Jennifer N. Dootz, Scott A. Jackson

**Affiliations:** 1 Biosystems and Biomaterials Division, NIST 536200 , Gaithersburg, Maryland, USA; University of Illinois Urbana-Champaign, Urbana, Illinois, USA

**Keywords:** compositionality, scaled abundance, internal standard, experimental design, metagenomic sequencing

## Abstract

The increasing use of metagenomic sequencing (MGS) for microbiome analysis has significantly advanced our understanding of microbial communities and their roles in various biological processes, including human health, environmental cycling, and disease. However, the inherent compositionality of MGS data, where the relative abundance of each taxon depends on the abundance of all other taxa, complicates the measurement of individual taxa and the interpretation of microbiome data. Here, we describe an experimental design that incorporates exogenous internal standards in routine MGS analyses to correct for compositional distortions. A mathematical framework was developed for using the observed internal standard relative abundance to calculate “Scaled Abundances” for native taxa that were (i) independent of sample composition and (ii) directly proportional to actual biological abundances. Through analysis of mock community and human gut microbiome samples, we demonstrate that Scaled Abundances outperformed traditional relative abundance measurements in both precision and accuracy and enabled reliable, quantitative comparisons of individual microbiome taxa across varied sample compositions and across a wide range of taxon abundances. By providing a pathway to accurate taxon quantification, this approach holds significant potential for advancing microbiome research, particularly in clinical and environmental health applications where precise microbial profiling is critical.

**IMPORTANCE:**

Metagenomic sequencing (MGS) analysis has become central to modern characterizations of microbiome samples. However, the inherent compositionality of these analyses, where the relative abundance of each taxon depends on the abundance of all other taxa, often complicates interpretations of results. We present here an experimental design and corresponding mathematical framework that uses internal standards with routine MGS methods to correct for compositional distortions. We validate this approach for both amplicon and shotgun MGS analysis of mock communities and human gut microbiome (fecal) samples. By using internal standards to remove compositionality, we demonstrate significantly improved measurement accuracy and precision for quantification of taxon abundances. This approach is broadly applicable across a wide range of microbiome research applications.

## INTRODUCTION

Over the last several decades, the decreasing cost and increasing throughput of next-generation sequencing (NGS) measurements have made metagenomic sequencing (MGS) characterization a default strategy for microbiome analyses (1–3). Owing to the ubiquity of naturally occurring microbiomes throughout natural and man-made environments, MGS analyses have correlated microbiomes, and their resident microbes, with a variety of important phenomena, including human, animal, and plant health, renewable resources, infrastructure degradation, environmental biogeochemical cycling, and waste remediation, among others (3–12). Particularly in the field of health, many connections have been postulated between the human microbiome and various health and disease conditions, such as obesity, gut health, autism, depression, or autoimmune disease (4, 6, 8, 13–18).

However, despite promising initial indications, many of the earliest correlations between microbiomes and health conditions have failed to bear fruit. For instance, questions have arisen about the relevance of the Firmicutes:Bacteroides ratio to obesity and gut health, about the existence of a “cancer microbiome,” and about the role of microbes in Autism (19–22). Even early in the Human Microbiome project, it was widely recognized that MGS results were much more reproducible (precise) than they were accurate (reflecting the underlying biology) (23, 24). Many comparisons have shown that analysis results vary significantly between samples and methodologies (25–30).

One of the complications with drawing conclusions from MGS results lies in the data’s inherent compositionality (25, 27, 30, 31). In short, the observed relative abundance of each taxon in a sample depends on both its actual abundance as well as the abundances of all other taxa in the sample. While aggregate compositional differences between samples can be accurately assessed, this severely limits the quantification of variations in the individual abundances of constituent taxa. Alternately, rigorous compositional data analysis strategies hold great promise for quantifying individual microbiome taxon abundances, correlating observed and actual abundances of constituent taxa, and allowing direct comparisons between microbiome samples with varied compositions (32).

A variety of ratiometric analysis strategies have been applied in the context of microbiome MGS to try to correct for data compositionality (32–35). One effective strategy has been to use ratios of pairs of taxon relative abundances within each sample to correct for the effects of compositionality (25, 36). Although this accurately accounts for compositionality, ratios of abundances of native taxa can be challenging to interpret and require that both taxa be present in all samples of interest. In wastewater biosurveillance for extracellular antimicrobial resistance genes by MGS, the routine addition of exogenous DNA as an internal standard has been demonstrated to improve MGS quantitation (37, 38).

In the current effort, we describe an experimental design for the routine inclusion of internal standards in microbiome MGS analyses. When genetic material from exogenous microbes is systematically added to samples, it serves as an internal reference standard to assess and mathematically correct for each sample’s composition. A rigorous framework for this analysis is provided along with several demonstrative examples. A previously reported systematic study of mock communities is reanalyzed here using the proposed internal standard framework. Then, a series of human gut microbiome samples are prepared and evaluated for two demonstration cases: (i) constant microbe concentrations with varied sample compositions; and (ii) systematically varied microbe concentrations with constant sample composition. In all cases, the results validate the proposed mathematical model for using internal standards to improve quantitative MGS analyses.

## RESULTS

### Internal standard and mathematical framework

**Fig 1:**
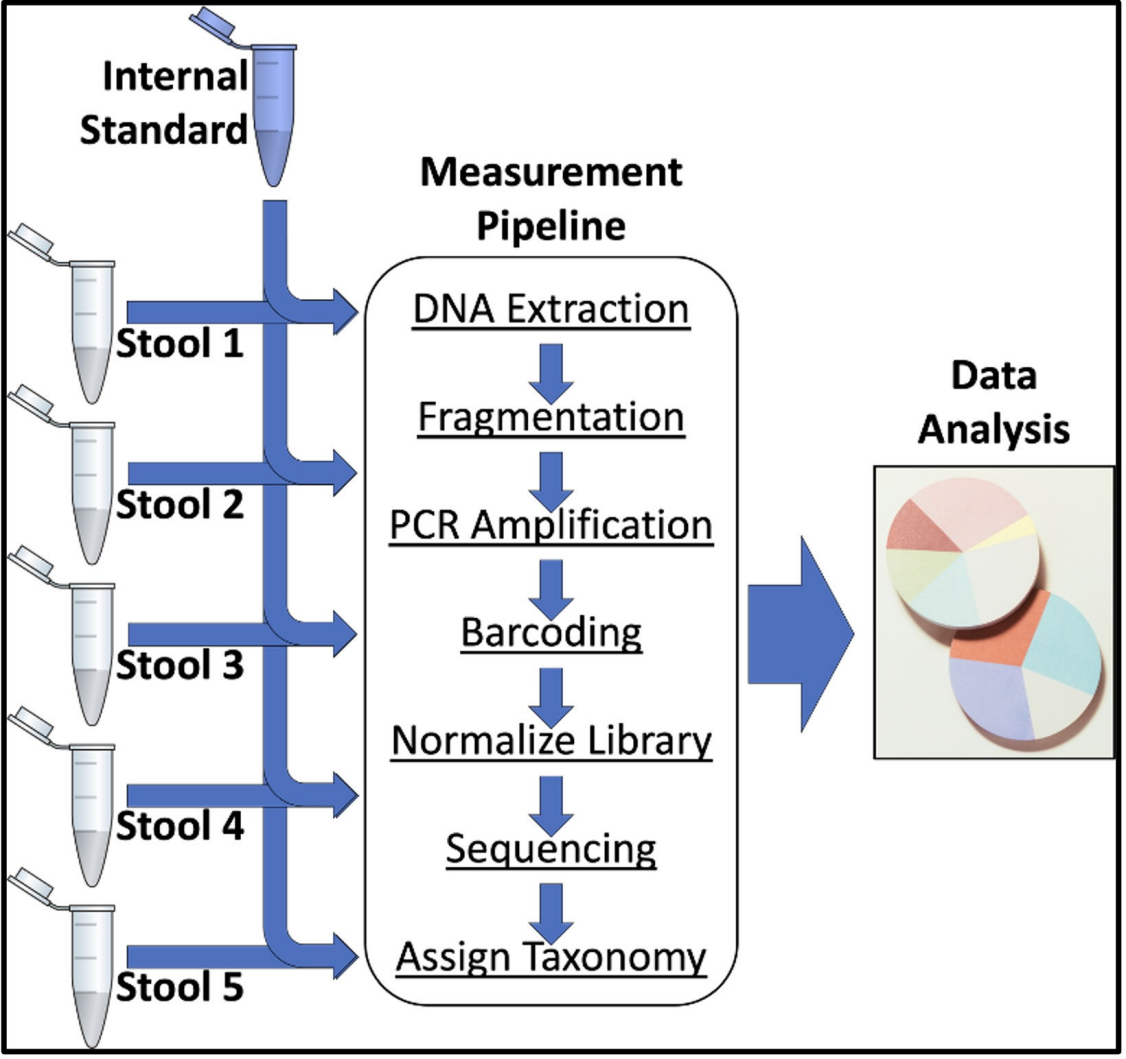
Experimental design specifying systematic addition of an exogenous internal standard microbe into every sample ahead of metagenomic sequencing analysis.

In the current effort, we propose an experimental design where an exogenous microorganism is systematically added into each sample to be analyzed as an internal standard (Fig. 1).

Following the inclusion of an internal standard, MGS results were analyzed to remove compositionality by comparing the relative abundance of each taxon to that of the internal standard. A mathematical framework was derived to use analysis of internal standards to improve MGS data (see Materials and Methods). We defined a new ‘Scaled Abundance’ metric that is calculated for each taxon using measured relative abundances and the known actual abundance of the internal standard (equation 4). These Scaled Abundances were mathematically predicted to be directly proportional to each taxon’s actual (biological) abundance, with a constant of proportionality based solely on the biases of that taxon and the internal standard (equation 5). Additionally, when a taxon of interest was present across multiple samples (analyzed using a common protocol), the bias ratio term remained constant, and the fold change in Scaled Abundance (ΔSA) was predicted to directly report the fold change in actual abundance (equation 6). The remainder of this manuscript demonstrates the validity and utility of equations 5 and 6 through a series of new analyses and experimental explorations.

### Previously published data

**Fig 2:**
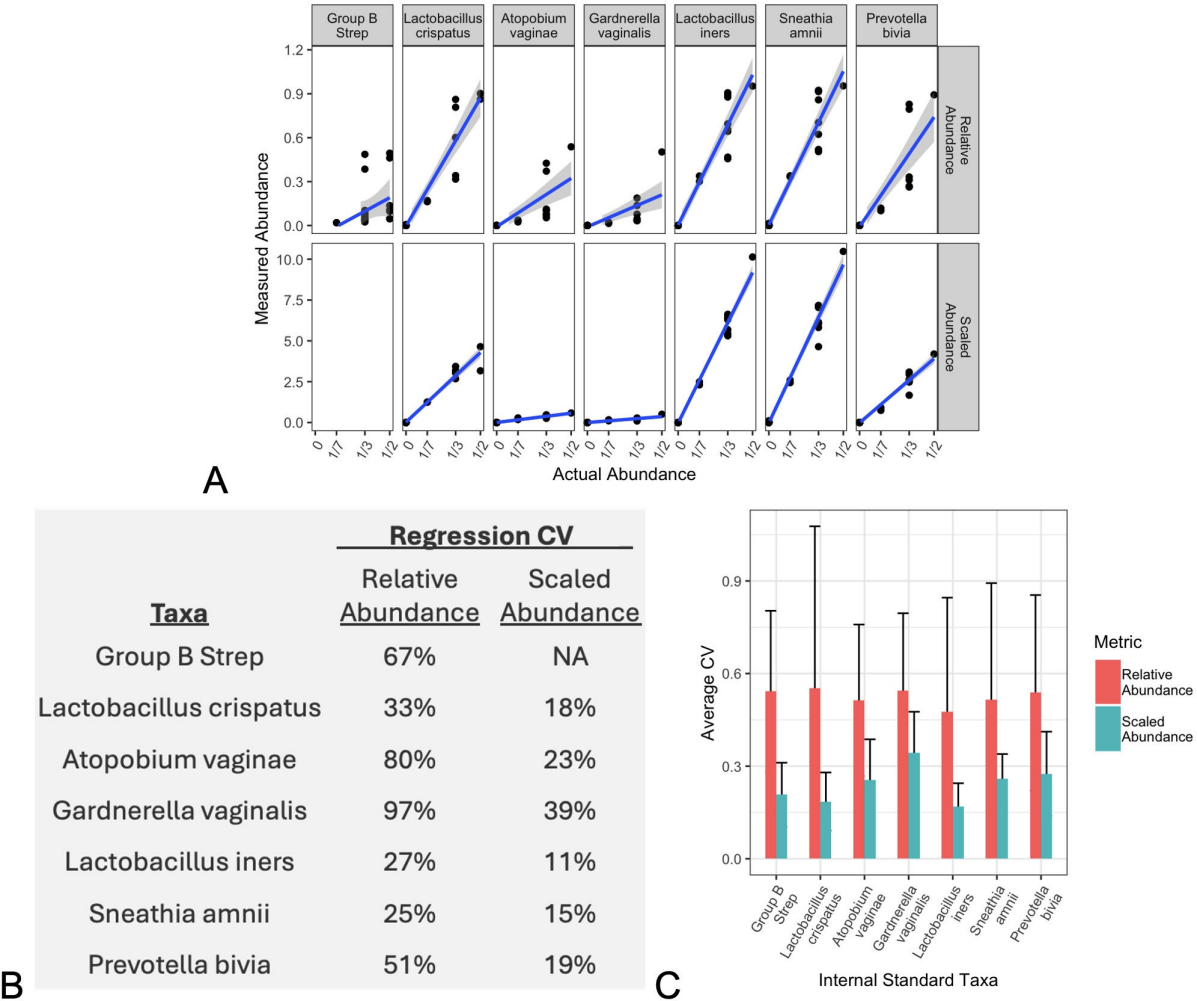
Previously published data from a series of microbial mock communities (26) were reanalyzed using the mathematical framework described herein by treating one strain as an internal standard (IS) for calculating Scaled Abundances (SAs). Strain abundances, as represented by relative abundance (RA) or SA, were plotted against their experimentally specified actual abundances (**A**). (Group B Strep was treated as the IS, so no SAs were calculated.) Linear regressions and 95% confidence bounds show best fit linear correlation models. The regression goodness-of-fits are summarized (**B**) as calculated coefficients of variation (CVs) for each regression. The Scaled Abundance for Group B Strep was not available (“NA”) because it was treated here as the internal standard. This procedure was generalized by systematically treating each strain as the IS, and calculating the average goodness-of-fit (and 95% confidence intervals) for remaining strains as measured by each metric (**C**).

In 2015, Brooks et al. systematically generated a series of 80 combinations of seven specific bacterial strains commonly associated with the vaginal microbiome (26). These diverse ‘mock community’ metagenomic samples were then analyzed using a common measurement pipeline, including DNA extraction, PCR amplification of the V1–V3 region of the rRNA gene, and NGS. We have reanalyzed this data set in the current framework by designating one added strain as an internal standard and using its specified abundance to calculate Scaled Abundances for the remaining taxa (Fig. 2).

As reported in the analysis from the original manuscript, measured relative abundance of individual taxa exhibited poor correlation with the actual abundances known from sample preparation (Fig. 2A, relative abundance). However, when we designated one of the taxa (e.g., Group B Strep) as an internal standard to correct for compositionality, the calculated Scaled Abundances for the remaining taxa were much better correlated with the known actual abundances (Fig. 2A, Scaled Abundances). Regressions for each taxon’s Scaled Abundance demonstrated direct proportionality with their actual abundances, as predicted by equation 5. The slopes of the Scaled Abundance regressions also varied between taxa, consistent with varying constants of proportionality in each case, as predicted by the bias ratios in equation 5.

For each taxon, the degree of agreement between the observed and actual abundances (goodness-of-fit) was summarized using a calculated coefficient of variation (CV, Fig. 2B). Using Group B Strep as the internal standard, the CVs for relative abundance measurements were quite large and consistently higher than the CVs for Scaled Abundances, presumably because the Scaled Abundance corrected for sample compositionality. Recognizing that any of the mock community taxa could have been treated as the internal standards, these analyses were repeated for each taxon used in the study. In each case, the average of CVs from the remaining taxa showed that Scaled Abundances, calculated using any taxa as the internal standard, consistently produced better correlation (lower CVs) between measured and actual abundances than relative abundances (Fig. 2C).

### Testable Hypothesis 1: Scaled Abundance is independent of sample composition

**Fig 3:**
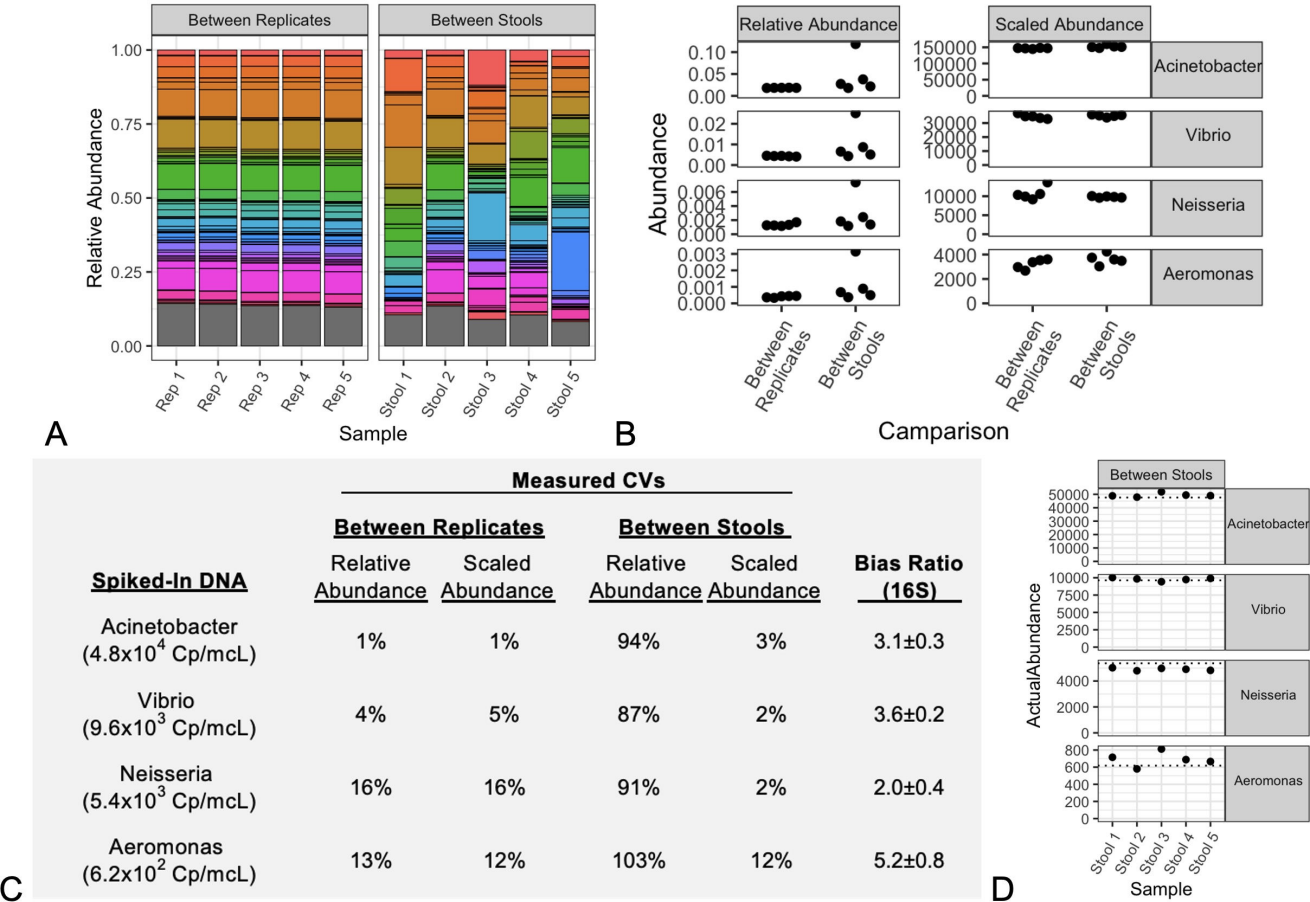
Metagenomic sequencing of diverse stool samples. Stacked barcharts (**A**) of the most abundant genera (colors) reveal high reproducibility between replicate analyses of a single stool sample, and significant compositional differences between stool samples from different donors. Known concentrations of DNA from four taxa were uniformly spiked-in, and their individual abundances (**B**) are plotted for the relative abundance (RelAbund) and Scaled Abundance (SA) metrics and grouped for comparisons between replicates and between distinct stools. The precision of these abundance measurements is tabulated (**C**) as calculated CVs. Data from the SA technical replicates were used to calculate bias ratios (±95% confidence interval), as described in equation 5. Using these bias ratios, the actual abundances for the “between stools” data were calculated from equation 5 and plotted (**D**) to show good correlation with ground truth spike-in concentrations (dotted lines). Shown here for 16S MGS analysis, the same samples were analyzed using a separate shotgun MGS analysis pipeline with substantially similar results (Fig. S1).

In order to demonstrate that the measured Scaled Abundances were independent of sample composition, a series of samples were prepared wherein constant amounts of microbial DNA from four exogenous taxa were spiked into individual fecal samples exhibiting identical compositions (i.e., technical replicates) or varied compositions (Fig. 3). The concentrations of the spiked-in DNA were systematically varied by taxa across two orders of magnitude, and they were then uniformly added to each sample. For each sample, MGS analysis yielded relative abundance measurements which were used to calculate Scaled Abundances for the spiked-in taxa through comparison to an internal standard as described in equation 4.

As expected for MGS analysis of fecal samples, good reproducibility was observed between replicate analyses, while significant compositional differences were observed between five different stool samples (Fig. 3A). However, when individual spike-in taxa were uniformly added to all samples, their measured relative abundances (Fig. 3B, RelAbund) were only consistent within a common sample composition. Even though the spiked-in DNA was known to be constant, the observed relative abundances between different fecal samples exhibited significant variation between different sample compositions. In contrast, Scaled Abundances calculated using an internal standard (Fig. 3B, ScaledAbund) exhibited consistency for both technical replicates and across differing sample compositions.

The precision of observed abundances (by relative abundance and Scaled Abundance) was calculated as the CV across each group of samples and for each of the spiked-in taxa (Fig. 3C). Within the measured relative abundances of technical replicates, CVs ranged 1–16%, with lower precision (i.e., CV > 10%) generally observed with lower spike-in concentrations. However, much higher variability in measured relative abundance (90–100% CV) was observed when taxa were spiked into varied sample compositions. In contrast, the calculated Scaled Abundances exhibited generally high precision (CV ≤ 16%) for all spiked-in taxa, both within technical replicates and between different sample compositions.

### Accounting for bias

While the calculated Scaled Abundances succeed at accounting for differences between sample compositions, they are not without bias. Indeed, equation 5 predicts that the constant of proportionality between the Scaled Abundances for each taxon and their Actual Abundances arises from the ratio of the analytical biases between the specified taxa and the internal standard. Using the known Actual Abundances of each of the spike-in taxa, bias ratios $\left(Bias_{Spike\;In}:Bias_{Internal\;Standard}\right)$ were calculated from the technical replicate fecal samples (Fig. 3C). While the measured biases are expected to be highly protocol-specific, they were highly reproducible across replicate samples for a specified protocol. These bias ratios were then used to calculate actual abundances from the Scaled Abundance measurements for each distinct stool sample (Fig. 3D), and these calculated absolute abundances exhibited excellent agreement to ground truth spike-in amounts (dotted lines in Fig. 3D).

**Fig 4:**
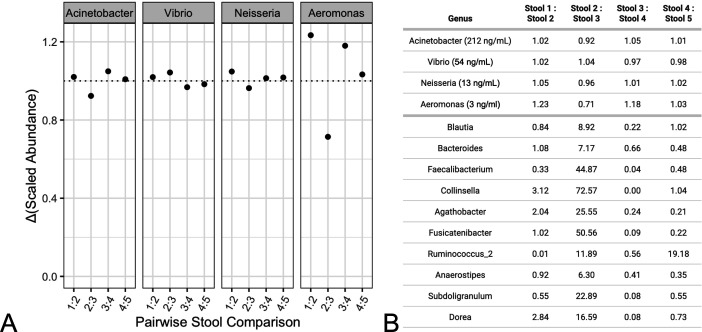
Δ(ScaledAbundance) for each of the spike-in taxa was calculated and plotted (**A**) for pairs of the stool samples shown in Fig. 3. Since the spike-ins were uniformly added to all samples, Δ(ScaledAbundance) was expected to be unity (dotted line). The table (**B**) provides calculated Δ(ScaledAbundance) for each of the spike-in taxa and the top 10 most abundant taxa quantified in all five stool samples. Shown here for 16S MGS analysis, the same samples were analyzed using a shotgun MGS analysis pipeline with substantially similar results (Fig. S2 and S3). An exhaustive list of all taxa and their measured Δ(ScaledAbundance) values are also available (Table S1).

Alternately, Δ(ScaledAbundance) was calculated by comparing Scaled Abundances between pairs of fecal samples, as described by equation 6. For the uniformly added spike-in taxa, the results hovered around unity for all of the uniformly added taxa and samples considered (Fig. 4A). The calculated Δ(ScaledAbundance) for the spike-in taxa as well as the most abundant 10 native taxa are also tabulated (Fig. 4B). For native taxa, the fold changes varied significantly between pairs of samples, as expected for diverse fecal samples.

When the same fecal samples were analyzed using shotgun MGS (instead of 16S MGS), substantially similar results were attained (Fig. S2). In particular, the quantified Δ(ScaledAbundance) values for native taxa between samples were nearly identical (Fig. S3 and Table S1).

### Testable Hypothesis 2: Scaled Abundance is proportional to actual abundance

**Fig 5:**
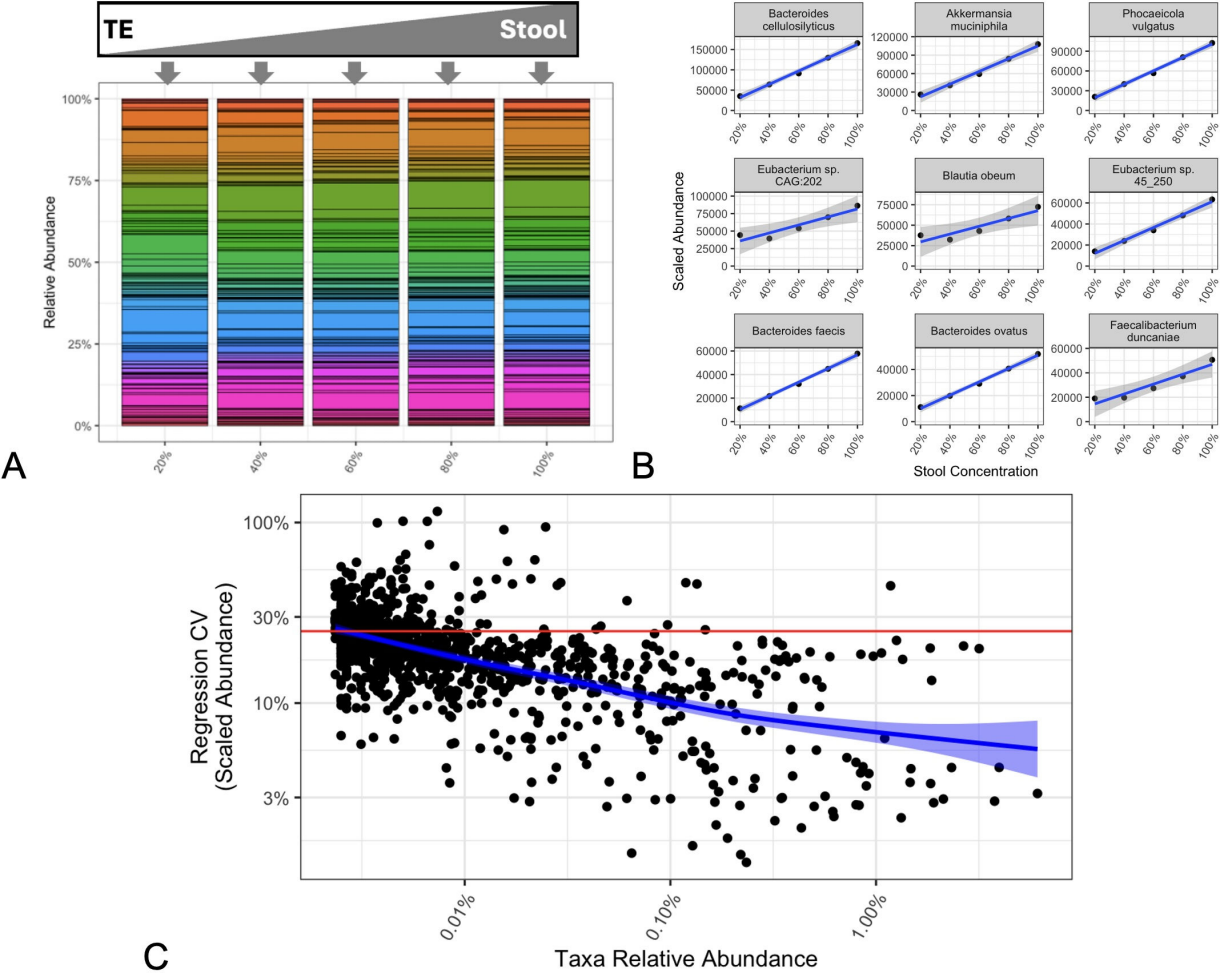
Metagenomic sequencing of a human stool dilution series. Stacked bar charts (**A**) of the most abundant species (colors) for a systematic dilution of a single stool sample with TE buffer show a consistent sample composition. Regression showed that measured Scaled Abundance and actual stool concentration (**B**), for the nine most-abundance native taxa) were highly correlated. (Relative abundances for the same taxa exhibited poor correlations with stool concentration, as seen in Fig. S4.) Similar regressions were determined for all taxa native to the stool, and their goodness-of-fits were plotted (**C**) as a function of each taxon’s measured relative abundance. Across all taxa (blue smoothed fit, 95% confidence interval), these regressions revealed high correlation (CV ≤ 25%, horizontal red line) between measured Scaled Abundances and known actual abundances across a wide range of taxon relative abundances.

To further demonstrate the utility of an experimental design that includes internal standards, a series of samples were prepared wherein the sample composition was kept constant even while microbial abundances were systematically varied. This was accomplished by diluting a single fecal sample into Tris-EDTA buffer (Fig. 5A). As can be seen from the stacked bar charts of taxon relative abundances, as measured by shotgun MGS, sample composition remained unchanged across all samples, even while the Actual Abundances of native taxa were known to be decreasing based on sample preparation. This dilution series was then treated as a series of independent samples, into which the internal standard was uniformly added. For these samples, the dilution fraction served as a stand-in for taxon actual abundance when comparing between samples, and measured abundances were correlated with dilution fraction as described in equation 2a and equation 2b (relative abundance) and equation 5 (Scaled Abundance).

When individual Scaled Abundances of the most abundant native taxa were compared to each sample’s known fecal concentration, good correlations were observed, as predicted from equation 5 (Fig. 5B). In contrast, analysis of relative abundances for the same taxa produced poor correlations, which were generally indistinguishable from the null hypothesis of no correlation (Fig. S4).

While regressions only from the nine most abundant taxa in the stool sample are shown, linear fits of Scaled Abundance to fecal concentration were calculated for each native taxa, the goodness-of-fit was measured for each regression as a CV, and these CVs were plotted as a function of taxon relative abundance in whole stool (Fig. 5C). Across a wide range of taxon abundances (down to a relative abundance of 0.00003), the calculated Scaled Abundances were generally (blue trace in Fig. 5C) accurate in reporting changes to actual taxon abundances with high precision (CV ≤ 25%).

## DISCUSSION

### Internal standard and mathematical framework

Because the protocols used in MGS analyses vary substantially between research groups, it can be challenging to address specific measurement biases in ways that are generalizable. However, the strategy of incorporating internal standards into samples of interest (Fig. 1) allows direct correction for the particular biases of the protocol being used. Internal standards have been advantageously employed for other analytical methods such as fluorescence quantitation, gene arrays, and metabolomic analyses to correct for method- and sample-specific biases (39–41). For application in MGS data sets, the internal standard was readily distinguished from other taxa based on its unique genome sequence, and its measured relative abundance was used to correct for compositional distortion.

The analysis described herein focuses on a measurement pipeline that is held constant between samples. The mathematical model (described in Materials and Methods) explicitly assumes that each step of the MGS measurement pipeline (e.g., DNA extraction, library preparation, NGS, read trimming, and taxonomic assignment) contributes biases that vary between microorganisms and accumulate throughout the protocol steps, but remain constant between multiple samples analyzed within a locked-down (i.e., fully specified) method (25, 30). Thus, these individual biases could be aggregated using a single taxon-specific bias term, as shown in equation 1 and equation 2a and equation 2b (25).

The process saturation that leads to the denominator in equation 2a and equation 2b explicitly denotes the inherently compositional nature of MGS data sets, since the measured relative abundance for each taxon is inextricably tied to the relative abundance of all other taxa within the sample. So, for example, a measured increase in one taxon’s relative abundance could arise from either an increase in its actual biological abundance or from a decrease in the actual abundances of other taxa.

These two scenarios are phenomenologically distinct but generally indistinguishable experimentally, particularly as (i) the actual abundances and biases remain unknown for most taxa in naturally occurring samples and (ii) biases vary substantially and unpredictably between individual taxa and various common protocols (25, 30). Thus, while whole-sample comparisons based on measured relative abundances generally reflect aggregate compositional changes with some accuracy, at least within the context of locked-down protocols, the quantitative comparison of individual taxon relative abundances between samples is unreliable. Instead, compositional data analysis approaches developed for or adapted to MGS data sets often take advantage of the constancy of the denominator in equation 2a and equation 2b among all taxa within a sample to remove or diminish compositionality using ratios, as is shown in equation 3 (25).

Using the systematically added internal standard for the taxon comparison improves on equation 3 by removing compositionality without the ambiguity of referencing two native taxa that may change independently of each other between samples. Then, a new metric, Scaled Abundance, is defined by explicitly comparing each native taxon relative abundance to that of the internal standard and accounting for the known internal standard actual abundance. The result of this experimental and mathematical approach is a direct proportionality between each native taxon’s calculated Scaled Abundance and its actual abundance, which is independent of sample compositionality (equation 5).

In the common case where multiple samples are analyzed using a common protocol, the bias ratio from equation 5 is constant across samples, and the fold change in a taxon’s Scaled Abundance between pairs of samples becomes independent of the bias ratio and directly reports the fold change in actual abundance (equation 6). This mathematical framework is further demonstrated through several test cases.

### Previously published data

The data from Brooks et al. was systematically collected, annotated, and made readily available, making this data set useful for reanalysis here (25, 26). For the purpose of evaluating the mathematical framework described here (see Materials and Methods section), we arbitrarily selected one of the strains added to various mock communities to treat as an internal standard with a known actual abundance in order to calculate Scaled Abundances for the remaining strains. The results for these Scaled abundances (Fig. 2) were entirely consistent with the predictions from equation 5, including improved correlation between measured and actual abundances, varied constants of proportionality (varied slopes between taxa in Fig. 2A, Scaled Abundance), and reduced variability overall among mock community compositions (Fig. 2B). Importantly for this reanalysis, it did not matter which strain was selected to be treated as an internal standard (Fig. 2C). Overall, this reanalysis demonstrated that using internal standards to calculate Scaled Abundances significantly improved MGS quantitation and the ability to accurately correlate individual taxon measurements with known actual abundances.

### Testable Hypothesis 1: Scaled Abundance is independent of sample composition

In the first testable hypothesis, exogenous microbial DNA was systematically added to diverse fecal samples and analyzed to demonstrate that calculated Scaled Abundances were substantially independent of the sample compositionality (Fig. 3). For this evaluation, the precision of technical replicates (“Between Replicates”; CV ≤ 16%) was interpreted as the fundamental precision limit of the method employed. However, for Scaled Abundances, similar reproducibility was achieved even between diverse stool samples for all spiked-in DNA. This demonstrated, as predicted from equation 5 (see Materials and Methods section), that Scaled Abundances were independent of sample compositionality and achieved high precision across at least two orders of magnitude, and even at low relative abundances (RA ~ 0.0005).

An important implication of the presented mathematical framework (see Materials and Methods section) is that the calculation of Scaled Abundances is largely method independent. (While the bias particular bias terms in adata set are unique, the framework overall is generalizable.) This is demonstrated here in the application of Scaled Abundances to improve quantitation for 16S MGS data from the previously published data set (Fig. 2) and for 16S MGS data for Stool samples (Fig. 3). The samples here were also analyzed with a completely distinct protocol for shotgun MGS with substantially similar results (Fig. S1). Under each of these three distinct protocols, the specific bias terms in equation 5 vary, but the mathematical framework remains generalizable to improve the correlation between measured Scaled Abundances and taxon actual abundances.

### Accounting for bias

Because taxon-specific biases arise from the particular protocol steps employed, the bias ratio in equation 5 was predicted to be constant within locked-down protocols, and the calculated Scaled Abundance metric was predicted to enable direct comparisons of abundances between samples, independent of differing sample compositions. There are two primary applications for using this Scaled Abundance metric. In the first scenario, taxa of interest may have been previously identified, isolated, and cultured. In this case, the native taxa of interest can be spiked into samples to allow calculation of the bias ratio in equation 5. This, in turn, allows the Scaled Abundance measured in unknown samples to be directly converted into accurately measured actual abundances. Using this approach bias, ratios for spiked-in DNA were calculated from technical replicates (Fig. 3C) and then used to calculate actual abundances across diverse stool samples with good agreement to known concentrations (Fig. 3D). Fig. S1C and S1D show similar analyses using shotgun MGS instead of 16S amplicon MGS.

However, for many native taxa, bias ratios cannot be independently determined for taxa of interest (e.g., unknown or unculturable taxa). Nevertheless, in this scenario, the biases remain constant between samples (analyzed using a common protocol), so fold changes in Scaled Abundance between samples still directly report the fold changes in actual abundance (as predicted in equation 6). This approach is highlighted in Fig. 4 and 5 ;(and Fig. S2, Table S1, and Fig. S3). As expected, the uniformly spiked-in DNA species exhibited no differences between pairs of stool samples (Fig. 4A), while native taxa demonstrated very different fold changes between the biologically distinct stool samples. Importantly, the quantified fold changes for all taxa were largely consistent even when comparing across 16S and shotgun MGS analyses (Table S1 and Fig. S3).

In both cases, this experimental design for the addition of internal standards and the mathematical framework to calculate Scaled Abundances provides a rigorous and quantitative strategy for directly comparing taxon actual abundances between samples in ways that help understand real changes in the underlying sample biology at the resolution of individual taxa.

### Testable Hypothesis 2: Scaled Abundance is proportional to actual abundance

In a second testable hypothesis, systematic dilutions of human stool into PBS were treated as a series of distinct microbiome samples. For this series, the composition was held constant (Fig. 5A), while the actual abundances of constituent taxa were systematically varied. While the true biological abundances of native taxa in the samples remain unknown, the overall stool concentration (20–100%) provided a reasonable surrogate for correlating taxon actual abundances with calculated Scaled Abundances.

As predicted from equation 5, (i) strong correlations were observed between individual native taxa and their Scaled Abundances, and (ii) linear regressions exhibited varied slopes, consistent with a taxon-specific bias ratio (Fig. 5B). Additionally, CVs for each regression provided taxon-specific assessments of the predictive value of the Scaled Abundances. These CVs revealed generally good precision (CV ≤ 25%) across more than three orders of magnitude in observed relative abundance and showed a low limit of quantitation (relative abundance ~ 0.00003) in the current study.

### Conclusions

This study introduces a new experimental design for improving the reliability of microbiome analyses by incorporating internal standards to account for compositional distortions inherent in MGS data. The mathematical framework developed herein demonstrates that the inclusion of an exogenous internal standard, whose actual abundance is measurable, allows for the calculation of taxon-specific Scaled Abundances” that are (i) independent of sample composition and (ii) directly proportional to actual biological abundances. This approach is consistent with prior ratiometric analysis strategies, with the added simplicity of accounting for compositionality through comparison to a single, well-characterized taxa systematically added at a known actual abundance.

For example, while conventional relative abundances can be used to detect compositional shifts among all taxa in aggregate and to categorize whole microbiome samples (e.g., case-control comparisons), the calculated Scaled Abundance described herein will enable researchers to evaluate individual constituent taxa and quantify changes in taxon actual abundances between samples. This ability to quantitatively interrogate individual taxa is central for hypothesis generation and understanding how microbial composition relates to observed microbiome function. In longitudinal studies with locked-down protocols, Scaled Abundances will enable accurate tracking of individual microbial abundance over time (e.g., following a defined intervention). Finally, by correcting for MGS compositionality, the framework described here opens new opportunities for quantitatively characterizing conserved sub-microbiomes found within larger, variable microbiome compositions (42).

Through rigorous analysis of previously published mock community data and freshly analyzed gut microbiome samples, we show that calculated Scaled Abundances outperform traditional relative abundance measurements in both precision and accuracy and demonstrate their potential for quantitative metagenomic profiling. Specifically, Scaled Abundances calculated for MGS characterization of mock communities agreed much better than raw relative abundances with taxon actual abundances across varied compositions. When exogenous taxa were spiked into fecal samples at known actual abundances spanning several orders of magnitude, the Scaled Abundance metric accurately reflected actual abundances and was wholly independent of sample composition. Furthermore, the use of internal standards allowed accurate comparisons of native taxa between samples, even when taxon-specific analytical biases could not be independently measured. Finally, high precision and accuracy were demonstrated by using Scaled Abundances to track actual abundances of native taxa across a series of stool dilutions, even for low-relative abundance taxa.

The results presented here validate the proposed experimental design and mathematical framework that uses routine, systematic addition of internal standards to microbiome samples to correct for compositionality in MGS analyses. This is particularly crucial in microbiome studies where variability in sample composition often complicates the interpretation of data. This approach is flexible and is shown to be applicable to both shotgun and amplicon-based sequencing methods, which further broadens its utility across a wide range of microbiome research areas. Moreover, by offering a pathway to accurate and reproducible abundance measurements at the level of individual taxa, this methodology could play a pivotal role in advancing microbiome research, particularly in clinical and environmental health contexts where precise taxon quantification is essential for modeling diverse biological phenomena.

## MATERIALS AND METHODS

### Mathematical framework

The methods of analysis of microbiome samples using MGS vary among researchers but invariably require a series of physical and bioinformatic manipulations, typically including: sample acquisition, DNA extraction, library preparation, and NGS, as well as read trimming and taxonomic assignment (1, 25, 29, 30). Importantly, each step contributes biases that vary between microorganisms and accumulate throughout the measurement pipeline (25, 30). For a locked-down method, where the procedure is tightly specified and highly reproducible, individual biases from multiple steps of an MGS protocol can be combined into a single aggregate bias term for each taxon that describes the general analytical response throughout the entire protocol (25):


(1) $$Analytical\;Response_{Taxa\;z}=Actual\;Abundance_{Taxa\;z}\times Bias_{Taxa\;z}$$

However, this analytical response is not directly observable from MGS results due to distortion arising from process saturation. Since NGS analyses arbitrarily limit the total number of DNA molecules that are analyzed, the measured relative abundance for each taxon depends on both the analytical response of that taxon (equation 1) and the aggregate responses of all taxa in the sample:


(2a) $$Relative\;Abundance_{Taxa\;z}=\frac{Analytical\;Response_{Taxa\;z}}{\sum {}_{Taxa}(Analytical\;Response_{Taxa})}$$

(2b) $$Relative\;Abundance_{Taxa\;z}=\frac{Actual\;Abundance_{Taxa\;z}\times Bias_{Taxa\;z}}{\sum {}_{Taxa}(Actual\;Abundance_{Taxa}\times Bias_{Taxa})}$$

Recognizing that the compositional denominator in equation 2a and equation 2b is identical for each taxon within a sample, the ratio of the relative abundances of two native taxa can be considered independent of the rest of the sample composition (25):


(3) $$\frac{Relative\;Abundance_{Taxa\;z}}{Relative\;Abundance_{Taxa\;y}}=\left(\frac{Actual\;Abundance_{Taxa\;z}}{Actual\;Abundance_{Taxa\;y}}\right)\times \left(\frac{Bias_{Taxa\;z}}{Bias_{Taxa\;y}}\right)$$

We proposed herein an experimental design where an exogenous microorganism was systematically added into each sample to be analyzed as an internal standard (Fig. 1). Thus, equation 3 was rearranged to calculate a ‘Scaled Abundance’ metric as the product of the internal standard actual abundance and the ratio of each native taxon’s relative abundance to the internal standard relative abundance.


(4) $$Scaled\;Abundance_{Taxa\;z}=\frac{Relative\;Abundance_{Taxa\;z}}{Relative\;Abundance_{Internal\;Standard}}\times Actual\;Abundance_{Internal\;Standard}$$

Algebraic manipulation of equations 3 and 4 then predicted that Scaled Abundances for each taxon would then be directly proportional to that taxon’s actual abundance, with a constant of proportionality tied to the ratio of the biases for that taxon and the internal standard:


(5) $$Scaled\;Abundance_{Taxa\;z}=Actual\;Abundance_{Taxa\;z}\times \left(\frac{Bias_{Taxa\;z}}{Bias_{Internal\;Standard}}\right)$$

Finally, since taxon-specific biases were assumed to be constant between samples (analyzed using a common protocol), changes in the Scaled Abundance of taxa of interest were predicted to directly report changes in taxon actual abundance without the need to calculate bias ratios:


(6) $$\Delta (Scaled\;{Abundance}_{Taxa\;z})=\Delta (Actual\;{Abundance}_{Taxa\;z})$$

### Mock community data analysis

Amplicon-based MGS analyses of 80 distinct mock communities of seven microbial taxa were downloaded from the original publication (26). The downloaded results provide relative abundances for each taxon in each mock community. These data were reanalyzed in R (version 4.3.2) here by treating “Group B Strep” as an internal standard to allow calculation of Scaled Abundances for the remaining taxa. (Mock communities that did not include Group B Strep were omitted from further analysis.) The specified dilutions from overnight stock (e.g., 1/2, 1/3, and 1/7) were taken as the known actual abundances for each taxon and correlated to measured relative abundances or Scaled Abundances. The goodness-of-fit of these regressions was summarized as CVs for each taxon. This procedure was repeated, treating alternate strains as the ‘internal standard’, and the average of CVs in each case was used to compare goodness-of-fits with respect to internal standard selection. The raw data and full code used for this analysis have been made publicly available (doi.org/10.18434/mds2-3760).

### Human microbiome sample prep

The human microbiome samples used in this study were used previously in the Mosaic Standards Challenge and have been described previously (29, 30). In short, five fecal samples were collected from five different anonymized donors. Each sample was prepared by pooling and homogenizing multiple bowel movements from each donor, stabilizing the mixtures with Omnigene Gut Solution, and preparing 1 mL identical aliquots at a final concentration of 100 mg/mL. Fecal aliquots were stored at −80 C until ready for analysis. Dilution series were generated by mixing the thawed samples into the Tris-EDTA buffer.

### Internal standard

The experimental design described here specified the systematic addition of an internal standard prior to the traditional MGS measurement pipeline. We selected DNA from *Legionella pneumophila* as an internal standard due to its absence in previous metagenomic characterizations of the stool samples. Additionally, this material was stable and had been well quantified by ddPCR previously (43). Genomic DNA from *L. pneumophila* was added uniformly to all samples for a final concentration of (2.16 × 10^5^ ± 3.7 × 10^3^) copies/mcL.

### Spike-in DNA

For experiments where specified concentrations of microbial DNA were spiked-in to fecal samples (i.e., Testable Hypothesis #1), pure genomic microbial DNA was added from *Acinetobacter baumanii, Vibrio furnissii, Neisseria meningitidis*, and *Aeromonas hydrophila*. These DNA genomes were sourced from NIST RM8376 (43) and were added to fecal samples at the final concentrations of 4.8 × 10^4^, 9.6 × 10^3^, 5.4 × 10^3^, and 6.2 × 10^2^ copies/mcL, respectively.

### MGS measurement pipeline

#### DNA extraction

DNA was extracted using the ZR Fecal DNA miniprep (cat# D6010) following the manufacturer’s protocol. Briefly, each sample was combined with 400 mcL of the lysis buffer and vortexed (MoBio Genie 2) for 20 min at full speed. Lysis tubes were centrifuged at 10,000 × *g* for 1 min, and the lysate was processed through the spin filter at 7,000 × *g* for 1 min. The filtrate was added to a 1.2 mL DNA binding buffer, mixed by pipetting, and centrifuged in batches at 10,000 × *g* through the spin column. The bound DNA was washed and eluted into 150 mcL of elution buffer. The extracted DNA was quantified by fluorescence using the DeNovix DS-11FX fluorometer with the DeNovix dsDNA High Sensitivity kit (Catalog # KIT-DADNA-HIGH-2).

#### Library preparation

NGS libraries were prepared for both shotgun and 16S amplicon analyses. For shotgun sequencing, extracted DNA was fragmented, amplified, and barcoded using the Nextera XT DNA Library Preparation Kit (Illumina) and Nextera XT Index kit V2 (Illumina, catalog #15052163) as specified by manufacturer protocols. For amplicon sequencing, the 16S rRNA gene was amplified using primers for the V4 variable region sourced from IDT (10 mcM RxnRead Primer Pool). 1mcL of the primer pool was combined with 12.5 mcL of Kapa HiFi HotStart readymix (KapaBiosystems, Catalog # 07958935001) and 12 ng extracted DNA in a final volume of 25 mcL for PCR (initial denaturation (180 s at 95°C), 18 cycles of denaturing (30 s at 98°C), annealing (15 s at 55°C), and elongation (20 s at 72.0°C), and a final extension (300 s at 72.0°C). The 16S amplicons were purified using SPRIselect beads (Beckman Coulter, Catalog # B23318) at a 0.8:1 ratio of Beads:amplicon, washing beads with molecular-grade ethanol and resuspended in pure water. Finally, barcodes were added (Nextera XT Index kit V2) as specified by manufacturer protocols. For both shotgun and amplicon library prep, samples were quantified by fluorescence (DeNovix) for DNA yield, and 10 ng from each sample was pooled for sequencing.

#### Sequencing

Pooled libraries were quantified by fluorescence, diluted to 4 nM, and denatured following manufacturer protocol (Document # 15039740 v10, Protocol A). Denatured libraries were further diluted to 12 pM, combined with a 5% PhiX (V3 cat# 15017666 from Illumina), and sequenced by paired-end sequencing on an Illumina MiSeq (MiSeq Reagent Kit v3 600-cycle, cat #: MS-102-3003).

### Initial bioinformatic analysis

Demultiplexing and adapter trimming was completed as part of the Illumina MiSeq Generate FASTQ workflow. Fastq files were analyzed separately for shotgun and amplicon sequencing, as described subsequently. The final output of both shotgun and amplicon bioinformatic analysis was a table specifying the taxa observed in each sample and their measured relative abundances.

For shotgun sequencing, BBduk (38.90) (44) was used to quality filter the raw data, and paired-end sample data were analyzed using Centrifuge (45) with the Web of Life database (46). The bash script files have been made publicly available (doi.org/10.18434/mds2-3760).

For amplicon sequencing, Cutadapt (2.8) (47) was used to remove primer sequences, and DADA2 (1.20.0) (48) was used to account for sequencing errors, determine absolute sequence variants, measure relative abundances, and assign taxonomy based on the Silva database (version 132) (49). The raw data and code (R, version 4.3.2) (50) used for amplicon sequencing analysis have been made publicly available (doi.org/10.18434/mds2-3760).

### Scaled Abundance calculations

The definitions and mathematical framework for calculating the Scaled Abundance metric from tables of identified taxa from each sample and their measured relative abundances is provided in the “Mathematical framework” section of Materials and Methods. These operations were implemented in R (version 4.3.2), and all raw data and the code have been made publicly available (doi.org/10.18434/mds2-3760).

## Data Availability

All raw data and analysis code described in this paper have been made publicly available (doi.org/10.18434/mds2-3760).

## SUPPLEMENTAL MATERIAL

Supplemental material aem.01126-25-s0001.pdf Figures S1 to S4; Table S1.

ASM does not own the copyrights to Supplemental Material that may be linked to, or accessed through, an article. The authors have granted ASM a non-exclusive, world-wide license to publish the Supplemental Material files. Please contact the corresponding author directly for reuse.
