# Supplementary material for: A mathematical framework to correct for compositionality in microbiome data sets
Source: Appl Environ Microbiol. 2026 Jan 6;92(1):e01126-25. doi: 10.1128/aem.01126-25 (PMC12889883; doi:10.1128/aem.01126-25)

Supporting Information for:

A mathematical framework to correct for compositionality in microbiome datasets

Authors: Samuel P. Forry, Stephanie L. Servetas, Jason G. Kralj, Monique E. Hunter, Jennifer N. Dootz

SI Figures and Tables included:

- ***Figure SI 1:*** *Metagenomic sequencing of diverse stool samples, by shotgun MGS*
- ***Figure SI 2:*** $\boldsymbol{\Delta}(ScaledAbundance)$*, by shotgun MGS*
- ***Table SI 1:*** *Exhaustive summary of* $\boldsymbol{\Delta}(ScaledAbundance)$ *for all taxa*
- ***Figure SI 3:*** Comparison of $\boldsymbol{\Delta}(ScaledAbundance)$ *for shotgun and amplicon MGS*
- ***Figure SI 4:*** *Comparisons of measured relative abundance to actual stool concentration.*

Figure SI 1: *Metagenomic sequencing of diverse stool samples: this figure shows analysis of the same samples as Figure 3 from the main manuscript, but with shotgun MGS analysis (instead of amplicon MGS). Stacked barcharts (A) of the most abundant genera reveal high reproducibility within replicate analyses of a single sample, and significant compositional differences between stool samples from different donors. The individual abundances of spiked-in microbial taxa (B) are plotted for the relative abundance and Scaled Abundance metrics and grouped for comparisons between replicates and between stool samples. The precision of these abundances are tabulated (C) as a calculated coefficients of variation (CVs) alongside the bias ratio calculated from the 'between replicates' data based on equation 4 and the known spike-in concentrations. Using these Bias Ratios, the actual abundances for the 'between stools' data are calculated (D) and correlate well with the expected abundance (dotted lines).*

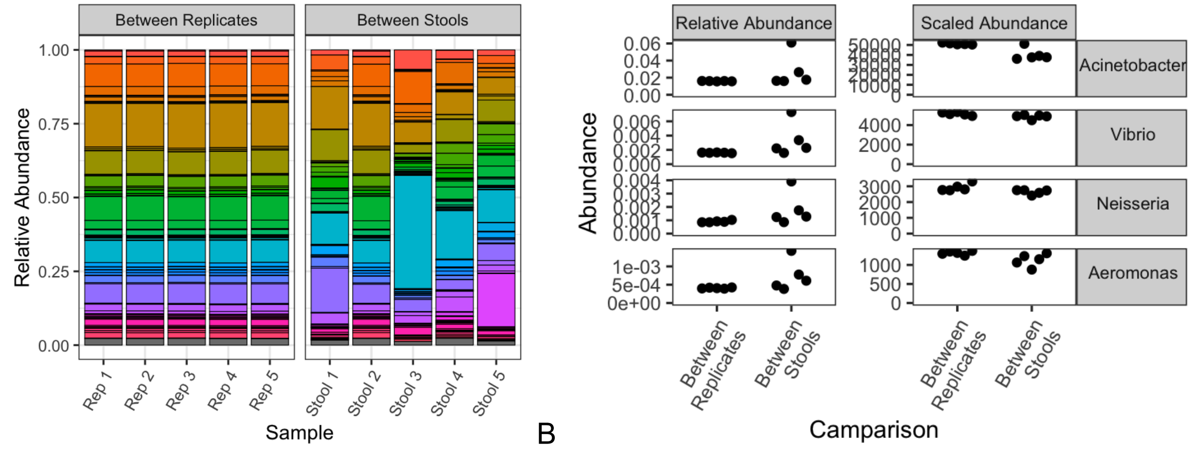

Spiked-In DNA	Measured CVs: Between Replicates — Relative Abundance	Between Replicates — Scaled Abundance	Between Stools — Relative Abundance	Between Stools — Scaled Abundance	Bias Ratio (WGS)
Acinetobacter ($4.8x10^4$ Cp/mcL)	1%	2%	70%	15%	10613±204
Vibrio ($9.6x10^3$ Cp/mcL)	3%	3%	69%	4%	4210±164
Neisseria ($5.4x10^3$ Cp/mcL)	8%	8%	57%	6%	9661±963
Aeromonas ($6.2x10^2$ Cp/mcL)	3%	4%	56%	15%	17356±819

C

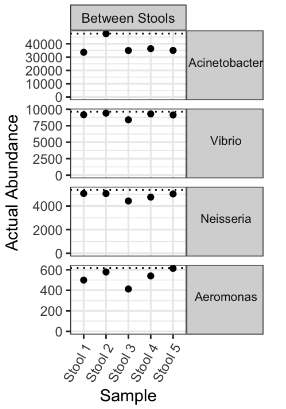

D

Figure SI 2: ***Δ(****ScaledAbundance), by shotgun MGS. This figure shows analysis of the same samples as Figure 4 from the main manuscript, but with shotgun MGS analysis (instead of amplicon MGS).* $\Delta(ScaledAbundance)$ *between pairs of stool samples was plotted (A) for each spike-in and agreed well with* $\Delta(ActualAbundance)$*. (Since the spike-ins were uniformly added to all samples,* $\Delta(ScaledAbundance)$ *should be unity in each comparison.) The table (B) provides calculated* $\Delta(ScaledAbundance)$ *for each of the spike-in taxa and the top 20 most abundant native taxa quantified in all 5 stool samples. Results are provided at the Genus taxonomic level to facilitate comparison to 16S results (Figure 4 of the main manuscript; the complete results for all taxa are tabulated (for both 16S and shotgun MGS) in Table SI 1 below.*

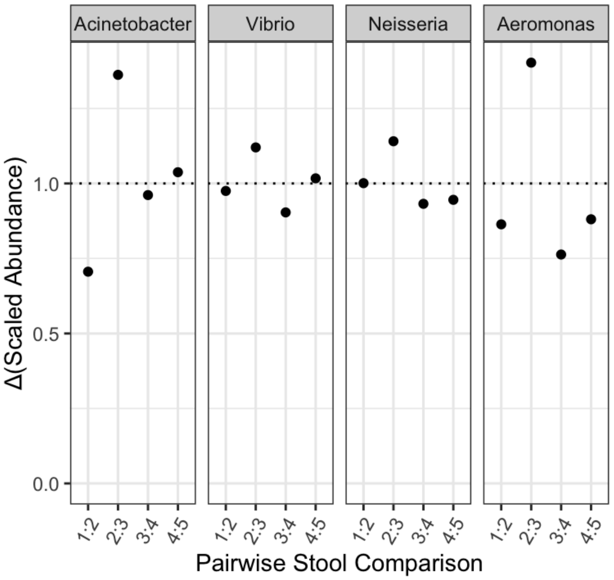

A

B

Genus	Stool 1 : Stool 2	Stool 2 : Stool 3	Stool 3 : Stool 4	Stool 4 : Stool 5
Acinetobacter (212 ng/mL)	0.71	1.36	0.96	1.04
Vibrio (54 ng/mL)	0.98	1.12	0.90	1.02
Neisseria (13 ng/mL)	1.00	1.14	0.93	0.95
Aeromonas (3 ng/ml)	0.86	1.40	0.76	0.88
Bacteroides	0.72	10.52	0.39	0.90
Blautia	0.94	13.51	0.17	0.70
Phocaeicola	1.68	7.91	0.51	0.40
Segatella	1.37	3.12	0.02	0.05
Akkermansia	0.04	3.48	126.42	0.55
Eubacterium	0.24	54.22	0.16	0.37
Alistipes	0.47	5.18	0.16	3.53
Clostridium	0.24	15.37	0.15	0.62
Faecalibacterium	0.40	26.30	0.06	0.76
Agathobacter	1.45	37.68	0.14	0.24
Roseburia	1.16	9.33	0.27	0.67
Ruminococcus	0.25	1.87	0.22	4.56
Mediterraneibacter	1.59	11.35	0.11	0.79
Collinsella	2.76	105.73	0.00	0.89
Parabacteroides	0.99	14.43	0.26	0.78
unclassified Bacillota genus	0.41	4.08	0.76	0.74
Dorea	2.31	11.07	0.12	0.85
Fusicatenibacter	1.08	31.52	0.10	0.38
Anaerostipes	0.91	6.23	0.36	0.54
Anaerobutyricum	2.56	1.39	1.72	0.20

Table SI 1*: Exhaustive summary of* $\Delta(ScaledAbundance)$ *measured between the 5 stool samples for all taxa called by both amplicon (16S) and shotgun (WGS) analyses. (This list expands the truncated lists shown in Figure 4 and Figure SI 2.) Genera are arranged in order of decreasing relative abundance in undiluted Stool.*

		Stool Comparison			
**Genus**	**MGS Type**	**1:2**	**2:3**	**3:4**	**4:5**
Acinetobacter	16S	1.02	0.92	1.05	1.01
	WGS	0.71	1.36	0.96	1.04
Vibrio	16S	1.02	1.04	0.97	0.98
	WGS	0.98	1.12	0.90	1.02
Neisseria	16S	1.05	0.96	1.01	1.02
	WGS	1.00	1.14	0.93	0.95
Aeromonas	16S	1.23	0.71	1.18	1.03
	WGS	0.86	1.40	0.76	0.88
Blautia	16S	0.84	8.92	0.22	1.02
	WGS	0.94	13.51	0.17	0.70
Bacteroides	16S	1.08	7.17	0.66	0.48
	WGS	0.72	10.52	0.39	0.90
Faecalibacterium	16S	0.33	44.87	0.04	0.48
	WGS	0.40	26.30	0.06	0.76
Collinsella	16S	3.12	72.57	0.00	1.04
	WGS	2.76	105.73	0.00	0.89
Agathobacter	16S	2.04	25.55	0.24	0.21
	WGS	1.45	37.68	0.14	0.24
Fusicatenibacter	16S	1.02	50.56	0.09	0.22

	WGS	1.08	31.52	0.10	0.38
Anaerostipes	16S	0.92	6.30	0.41	0.35
	WGS	0.91	6.23	0.36	0.54
Subdoligranulum	16S	0.55	22.89	0.08	0.55
	WGS	0.31	23.62	0.13	0.62
Dorea	16S	2.84	16.59	0.08	0.73
	WGS	2.31	11.07	0.12	0.85
Akkermansia	16S	0.08	4.10	198.87	0.35
	WGS	0.04	3.48	126.42	0.55
Lachnoclostridium	16S	7.08	1.24	1.63	0.29
	WGS	0.58	5.35	0.35	1.22
Alistipes	16S	0.29	4.64	0.24	2.86
	WGS	0.47	5.18	0.16	3.53
Serratia	16S	0.31	0.07	5.66	1.91
	WGS	0.19	13.26	0.30	0.86
Parabacteroides	16S	1.00	11.99	0.33	0.57
	WGS	0.99	14.43	0.26	0.78
Streptococcus	16S	10.07	0.91	0.13	2.46
	WGS	3.04	1.66	0.12	2.43
Marvinbryantia	16S	0.06	9.43	0.18	2.30
	WGS	0.57	1.90	0.90	1.48
Lachnospira	16S	0.83	6.37	0.93	0.24
	WGS	0.34	18.40	0.25	0.39
Roseburia	16S	0.21	11.41	0.13	0.85

	WGS	1.16	9.33	0.27	0.67
Butyricicoccus	16S	1.13	42.28	0.07	0.80
	WGS	0.87	22.17	0.11	0.90
Intestinibacter	16S	2.03	0.03	3.14	0.13
	WGS	1.82	0.34	2.34	0.16
Phascolarctobacterium	16S	1.04	123.04	5.73	0.30
	WGS	0.90	22.18	0.35	2.61
Adlercreutzia	16S	0.53	2.21	0.70	4.89
	WGS	0.40	2.74	0.68	2.57
Aliivibrio	16S	1.09	1.38	1.04	0.85
	WGS	1.03	1.35	1.04	0.80
Intestinimonas	16S	0.07	6.39	0.17	4.57
	WGS	0.17	7.28	0.18	1.93
Agrococcus	16S	0.99	1.34	1.05	0.90
	WGS	0.54	5.03	0.56	1.21
Methanobrevibacter	16S	0.00	337.73	5.84	0.81
	WGS	0.00	362.61	0.39	1.23
Oscillibacter	16S	0.18	17.05	0.10	3.79
	WGS	0.24	11.01	0.13	1.96
Hespellia	16S	1.43	3.54	0.54	1.54
	WGS	0.61	11.34	0.17	0.52
Flavonifractor	16S	0.57	9.91	0.07	0.48
	WGS	0.65	5.73	0.10	0.56
Turicibacter	16S	2.89	0.04	5.99	4.50

	WGS	0.91	0.43	2.55	1.58
Hungatella	16S	1.19	0.52	15.51	0.17
	WGS	0.63	4.01	0.50	0.91
Actinomyces	16S	5.57	1.33	0.09	4.99
	WGS	0.85	4.03	0.27	1.34
Holdemania	16S	0.29	19.32	0.25	0.59
	WGS	0.35	14.60	0.14	1.24
Dielma	16S	0.07	24.73	0.06	1.48
	WGS	1.17	1.48	0.88	0.35

Figure SI 3: *$\Delta(ScaledAbundance)$ between stool samples was determined independently for shotgun and amplicon MGS analyses and were strongly correlated. This supported the prediction of Equation 6 of the main text that $\Delta(ScaledAbundance)$ can be used to accurately quantify fold changes in taxa actual abundances, independent of analysis methodology.*

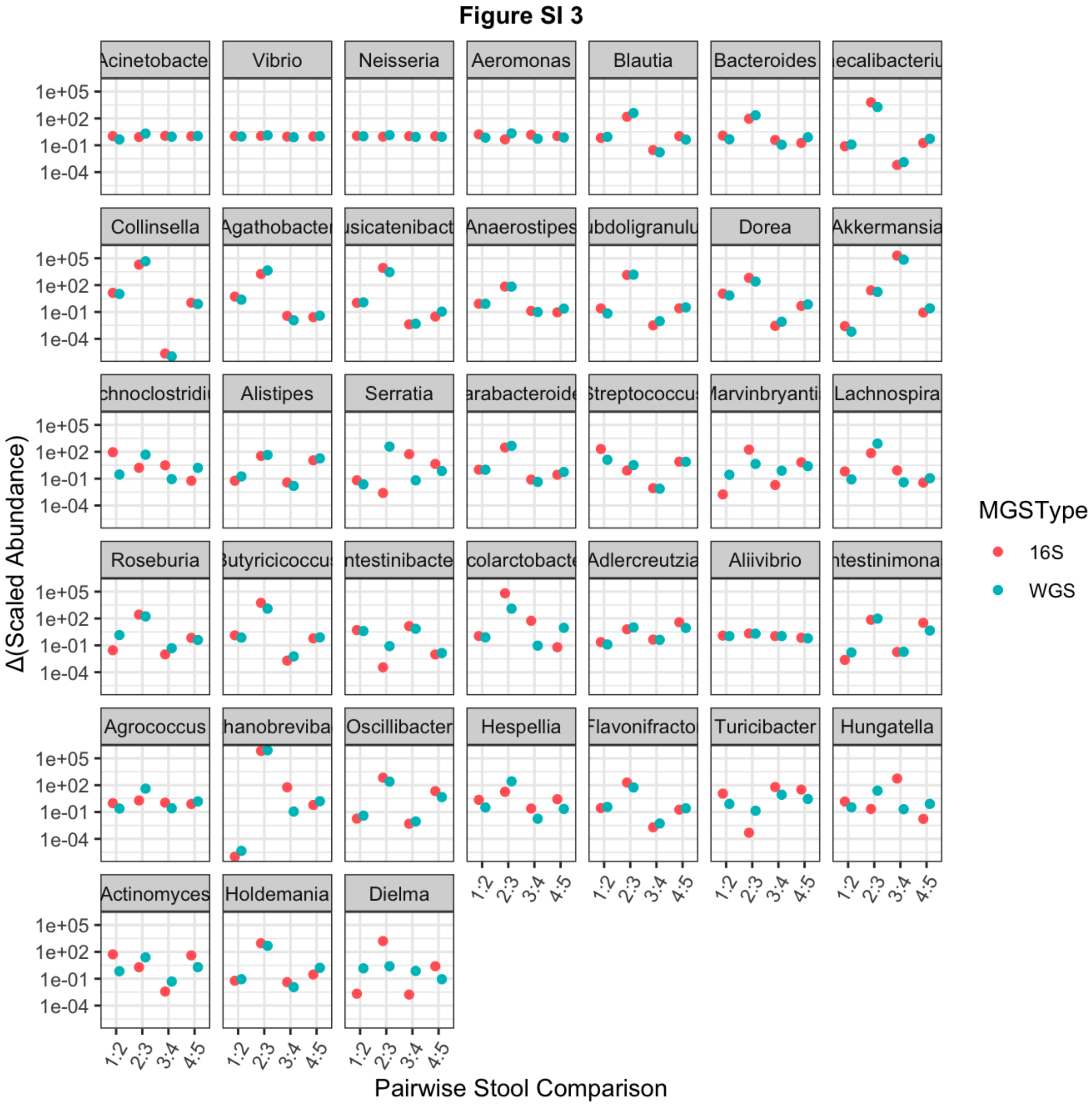

Figure SI 4: *Comparisons of measured relative abundance to actual stool concentration. As a corollary to Figure 5B from the main document, the observed relative abundances were plotted against stool dilution for each of the highest relative abundance taxa native to the stool. The linear regressions (and 95% confidence bounds) for each taxa were generally indistinguishable from the null hypothesis of no correlation.*

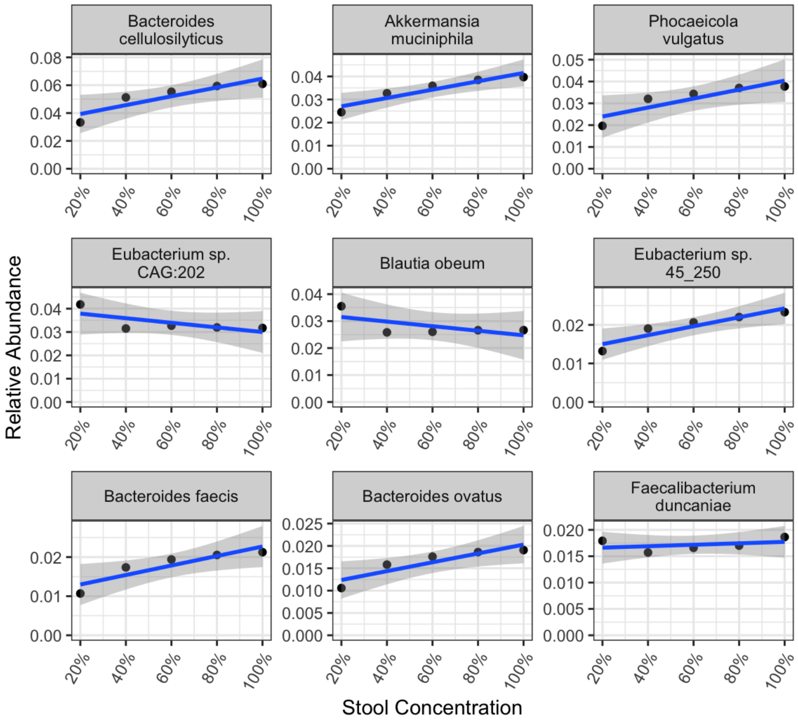

Supplement: Supplemental material — Figures S1 to S4; Table S1. [file aem.01126-25-s0001.pdf]
